# Developing an App by Exploiting Web-Based Mobile Technology to Inspect Controlled Substances in Patient Care Units

**DOI:** 10.1155/2017/3195369

**Published:** 2017-02-14

**Authors:** Ying-Hao Lu, Li-Yao Lee, Ying-Lan Chen, Hsing-I Cheng, Wen-Tsung Tsai, Chen-Chun Kuo, Chung-Yu Chen, Yaw-Bin Huang

**Affiliations:** ^1^Department of Pharmacy, Kaohsiung Medical University Hospital, Kaohsiung, Taiwan; ^2^School of Pharmacy, College of Pharmacy, Kaohsiung Medical University, Kaohsiung, Taiwan; ^3^Division of Medical Information, Kaohsiung Medical University Hospital, Kaohsiung, Taiwan

## Abstract

We selected iOS in this study as the App operation system, Objective-C as the programming language, and Oracle as the database to develop an App to inspect controlled substances in patient care units. Using a web-enabled smartphone, pharmacist inspection can be performed on site and the inspection result can be directly recorded into HIS through the Internet, so human error of data translation can be minimized and the work efficiency and data processing can be improved. This system not only is fast and convenient compared to the conventional paperwork, but also provides data security and accuracy. In addition, there are several features to increase inspecting quality: (1) accuracy of drug appearance, (2) foolproof mechanism to avoid input errors or miss, (3) automatic data conversion without human judgments, (4) online alarm of expiry date, and (5) instant inspection result to show not meted items. This study has successfully turned paper-based medication inspection into inspection using a web-based mobile device.

## 1. Introduction

Our hospital (Kaohsiung Medical University Hospital) is a medical center to implement in a center-pharmacy model same as others in Taiwan. During emergency situations, floor medications (including controlled substances, emergency medications, and IV drips) could save the delivery time from the pharmacy to patient care units; therefore, it is absolutely necessary to stock floor medications in these units including wards and physical examination rooms. Regardless of controlled substances or emergency medications, all medications in patient care units are “stock on loan” from the pharmacy. Therefore, these units should have proper management over items, amount, and expiry date of floor medications. On the other hand, pharmacists could regularly inspect the floor medications in each unit to ensure medications are stored properly [1]. According to Taiwan's laws/regulations and drug specific storage, there is a checklist when a pharmacist carries out an inspection. Examples include the following: (1) the controlled substances should be locked up in a specialized cabinet and should be handed over with care; (2) high-alert medications, such as potassium chloride or neuromuscular blocking agents, should be labeled clearly and stored separately with other medications; (3) light-sensitive medications should be stored in lucifugal containers; and (4) refrigerated medications should be stored at temperature between 2 and 8°C [2].

In our hospital, the designated pharmacists monthly inspected controlled substances in patient care units and then wrote down and recorded the inspection results on paper. As of December 2014, there were 53 units stocked with controlled substances; therefore, it was necessary to print 53 paper checklists before inspection. In addition to the need for 4 pharmacists to perform this regular work, the inspection paper results should be entered into a computer system and subsequently emailed to the personnel of inspected units for performance improvement. As a result, a lot of labor and resources have been allocated to perform this monthly routine work. The motivation of this study was to simplify this present process to enhance the efficiency of controlled substance inspection in patient care units.

As information technology (IT) has grown exponentially in the past few years, each business has been improving its workflow by adopting IT in order to keep competitive in respective markets. Hospital Information System (HIS) has been developed as the product of combining IT with healthcare. Among various IT fields, the most popular is the web-based mobile technology applying the Internet (www) with mobile devices (such as PDA, smartphone, and Tablet computer) [3]. The common device would be the smartphone. The advantage of web-based mobile technology is that users could turn data input/output from traditional paper record or PC data storage into web-based mobile mode. Time and geographical constraints can be broken, and data can be transferred and processed more quickly and instantly [4]. There are a lot of healthcare related Apps (applications) developed and used in different operation systems (Apple iOS, Android, Windows Phone, Symbian, etc.) for smartphones, so it is shown that this technology is fast growing and quite mature [5–10].

The purpose of this study is to use a web-based mobile technology to develop an App that pharmacists can use on smartphones to monthly inspect the controlled substances in each stock unit. We attempted to turn traditional paperwork into a convenient tool to increase the work efficiency.

## 2. Materials and Methods

### 2.1. Computational Methods and Related Tools

Firstly, we collected the paper inspecting forms and previous inspection experience from the pharmacists to design the items and functions of this App.

Secondly, we performed discussions with the system developers in our hospital to provide the App interface design to suit the smaller screen on mobile devices. Finally, after evaluating the web security and connectivity with the mobile devices platform in our hospital, we selected iOS 7 (https://www.apple.com/ios/) as the App operation system, XCode (https://developer.apple.com/xcode/) as the development tool, Objective-C (http://en.wikipedia.org/wiki/ObjectiveC) as the programming language, and Oracle v11g (http://www.oracle.com) as the database.

**Figure 1:**
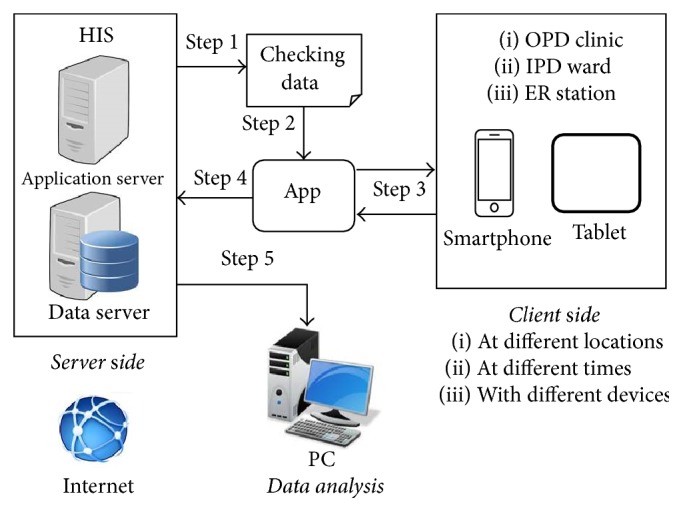
The mechanism of this App to inspect the controlled substances in patient care units.

The mechanism of this system is that our HIS for controlled substances management produces the monthly checking data from the database and these inspection results are instantly sent back to the HIS database through this App on the Internet in each inspected unit. Therefore, pharmacists are allowed to inspect the controlled substances with this App at different locations and at any time under this web-based IT configuration (Figure 1).

### 2.2. Item of Inspected Records and List of Controlled Substances

Our hospital is a medical center in southern Taiwan and included 1,667 beds, 545 physicians, and 119 pharmacists in 2014. The recording data of a total of 10 items contained drug name, drug amount, drug expiry date, specialized locked cabinet, high-alert medication management, lucifugal medication storage, refrigerated medication storage, drug storage location, drug labeling, and handover. All controlled substances in our hospital are listed in Table 1.

## 3. Results and Discussion

### 3.1. System Description

**Figure 2:**
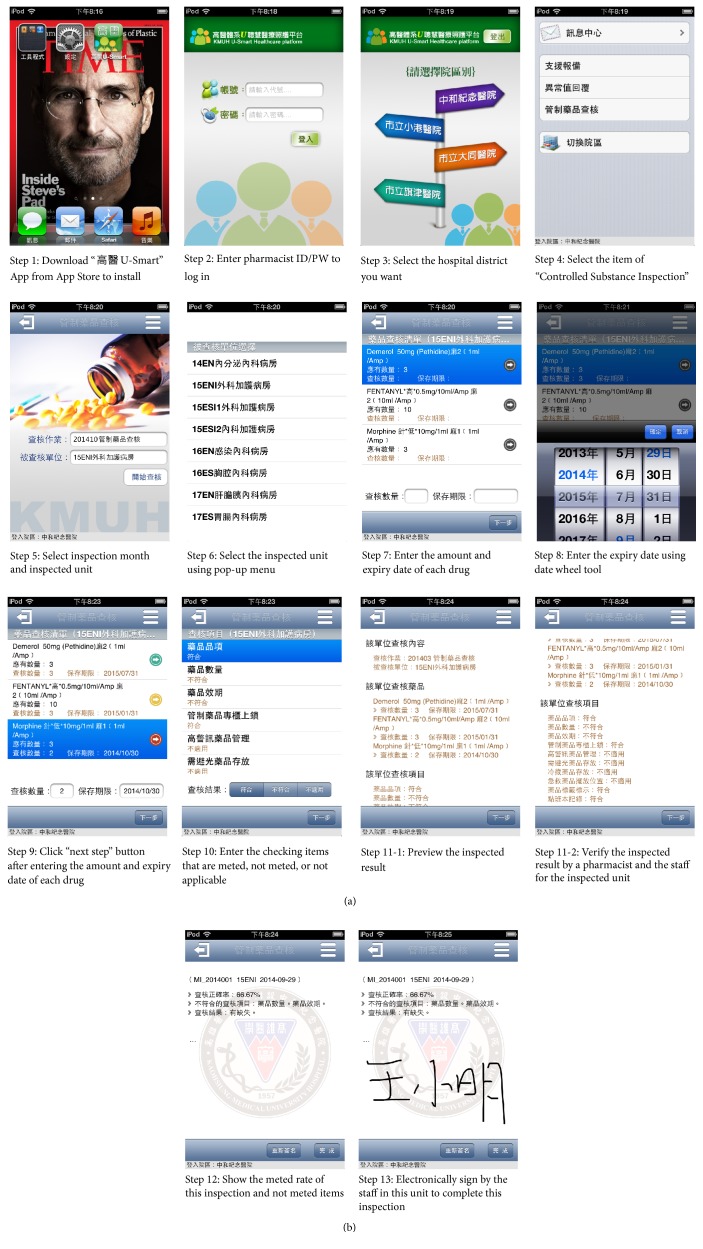
The introduction of how to operate the inspected App of controlled substances in this study (demo date: 2015/01/13).

This App was developed between July 2013 and August 2014 and has been shortly tested from September 2014 to December 2014. During the test, program errors were fixed and relevant training was provided to the staff. In January 2015, this App was officially launched for inspection and 2,128 records of inspection results (about 89 records in each month) through this App were added to our database until December 2016. The main steps of this App are (1) to select the inspection month and inspected unit after logging in with ID/PW; (2) to enter the amount and expiry date of each drug and the checking items; (3) to preview the inspection result and carry out verification by the pharmacist and the staff for the inspected unit; (4) to electronically sign to complete this inspection (Figure 2).

**Figure 3:**
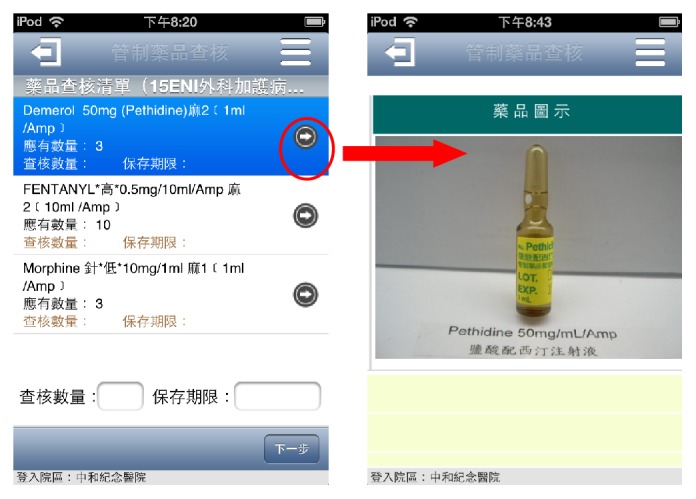
This App could display the latest drug appearance during inspection.

**Figure 4:**
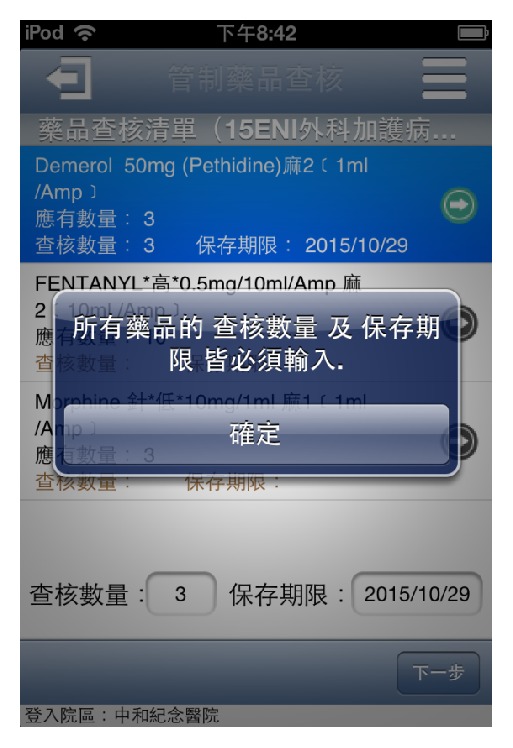
Required fields on inspection amount and expiry date.

This system not only is fast and convenient compared to the conventional paperwork, but also provides data security and accuracy. In addition, there are several features to increase inspection quality:

1. Accuracy of drug appearance: this App would display the latest drug appearance to find out the old package in patient care units during inspection (Figure 3).
2. Foolproof mechanism: this App supports error checking and assistant error messages to decrease input errors or misses (Figure 4).
3. Automatic data conversion: when the drug inspected amount (over or under) or expiry date (less than three months) does not meet inspection criteria, “Not meted” is automatically shown in the inspection result without human judgments (Steps  9 to 10 in Figure 2).
4. Online alarm of expiry date: when the expiry date is entered, if the period is more than six months, then this App would show a green light to indicate that this drug is safe to use. If the period is between three and six months, a yellow light is shown to indicate that this drug is close to expiry date and the staff in this unit should make an exchange with the pharmacy as soon as possible. When the period is less than three months, a red light is shown to indicate a not meted item (Step  9 in Figure 2).
5. Instant inspection result: before the result is electronically signed by the staff in this unit, this App automatically calculates the meted rate of this inspection (formula = number of meted items/(total items − not appropriate items) *∗* 100%) and shows not meted items to the staff. This instant inspection result makes the inspected unit immediately undergo performance improvement for these not meted items (Step  12 in Figure 2). The entire inspection information accessed by this App could be obtained by an Intranet application from a PC in our hospital.

### 3.2. System Requirements and Limitations

**Figure 5:**
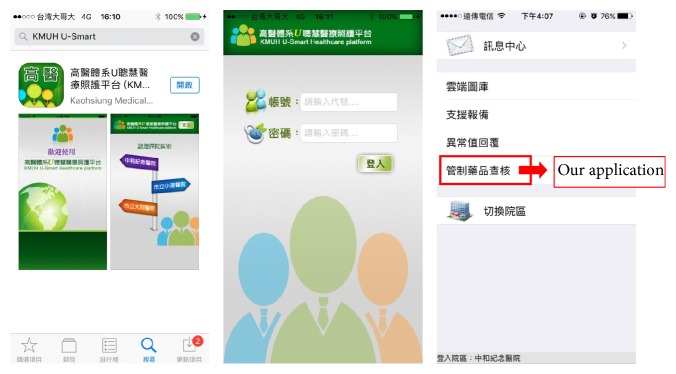
The KMUH U-Smart App and system application in the hospital.

This App is developed under Apple iOS 7 system; therefore, it is only supported on Apple-based mobile devices, such as iPhone, iPad, and iPod. This App is not installed in Android or Windows operating systems. In accordance with Apple launching iOS 8 in September 2014, this App was also instantly modified to be compatible on iOS 8 platform. The other limitation is that when the Internet was not stable, a few inspection results were not immediately recorded into the HIS database, and the pharmacist must manually enter this paper record into a computer system from a PC after inspection. Although this App does not apply patient data, it could be freely available (keyword search: KMUH U-Smart, Figure 5) and can be downloaded from the App Store to install and be used after the pharmacist logs in with the ID/PW in our hospital. This App can be easily used in other hospitals if they support web service between this App and its database including two tables to record four kinds of data: inspected project, inspected unit, drug information, and inspection result.

## 4. Conclusion

The trend to improve medication inspection now and in the future is speed and automation, and the key point depends on how to rapidly transfer data. Internet communication for data transfer is the best way to break time and geographical constraints, and it combines with mobile devices to provide a new model for medication inspection. This study has successfully turned paper-based medication inspection into inspection using mobile devices. Using a web-enabled smartphone, inspection can be performed on site and the inspection results can be directly recorded into the HIS through the Internet, so human error of data translation can be minimized and the work efficiency and data processing can be improved. Based on the successful start of this App, other types of floor medications (such as emergency medications) can be used with this model. This model can be also applied to other medication managements or other hospitals to construct a fast and automatic medication management platform.

## Figures and Tables

**Table 1:** List of controlled substances in our hospital.

	Oral	Injection	Topical use
1°	Morphine 10 mg/Tab Morphine SR 30 mg Tab Morphine XL 60 mg Tab	Morphine 10 mg/1 mL/Amp Morphine 20 mg/1 mL/Amp	Cocaine 6% sol

2°	Codeine 15 mg/Tab Methadone 10 mg/mL	Demerol 50 mg/1 mL/Amp Fentanyl 0.5 mg/10 mL/Amp Fentanyl 0.1 mg/2 mL/Amp	Durogesic 12 *μ*g/h/patch Fentanyl 25 *μ*g/h/patch

3°	Concerta ER 18 mg/Tab Concerta ER 36 mg/Tab Desud Plus 8 mg/Tab Modipanol 1 mg/Tab Ritalin 10 mg/Tab Temgesic SL 0.2 mg/Tab	Codeine 15 mg/1 mL/Amp Ketalar 500 mg/10 mL/Vial Temgesic 0.3 mg/Amp	
